# Parental acceptance of human papillomavirus vaccine for female adolescents in Abia State, southeastern Nigeria: a pre-implementation study

**DOI:** 10.11604/pamj.2025.51.44.43317

**Published:** 2025-06-13

**Authors:** Chidinma Ihuoma Amuzie, Amos Paul Bassi, Eliza Fishman, Dessie Mekonnen, Quail Rogers-Bloch, Kelli Cappelier, Scott LaMontagne, Kalu Ulu Kalu, Michael Izuka, Sola Thomas Sunday

**Affiliations:** 1 John Snow Incorporated, Abuja, Nigeria,; 2 Department of Community Medicine, Federal Medical Centre, Umuahia, Abia State, Nigeria,; 3 John Snow Incorporated Arlington, Virginia, USA,; 4 Nigeria Field Epidemiology and Laboratory Training Program, Abuja, Nigeria

**Keywords:** Adolescents, cervical cancer, human papillomavirus infections, human papillomavirus vaccines, Nigeria

## Abstract

**Introduction:**

cervical cancer is the second most common cancer amongst women in Nigeria, with an incidence rate of 26.2 per 100,000 and mortality rate of 14.3 per 1000 adult women. Vaccination is the primary prevention if initiated prior to Human Papillomavirus (HPV) infection. However, vaccine hesitancy remains a threat to the uptake of the HPV vaccine. This study identified the pattern and predictors of parental acceptance of HPV vaccines for their female adolescents in Abia State, Nigeria prior to state level introduction.

**Methods:**

we conducted a community-based cross-sectional study between August and September 2023 among parents of female adolescents residing in Abia State. A multistage sampling technique was used to select the study respondents. An interviewer-based, semi-structured questionnaire was administered to the respondents. Analysis was done using IBM SPSS version 26. Univariate analysis was used to present the socio-demographic characteristics of the respondents in frequencies and proportions. The association between parental acceptance and the independent variables was assessed using the chi-square test. Logistic regression was used to identify the independent predictors of parental acceptance of HPV vaccination. The level of significance was 5%.

**Results:**

a total of 1,016 respondents participated in this survey, with a mean age of 42.2 ± 10.5 years. The prevalence of parental HPV vaccine acceptance was 63.0% (95% CI: 59.8 - 66.0). The major sources of information on the HPV vaccine were the healthcare workers (43.0%) and social media (37.8%). Among respondents who would accept the HPV vaccine for their female adolescents, the commonest reason was for the prevention of HPV transmission (58.3%). Most of the respondents who declined willingness reported lack of information (63.9%), followed by fear of adverse effects (32.9%) as the triggers for non-acceptance of the vaccine. The most preferable source of HPV vaccine recommendation mentioned by the respondents were healthcare workers (92.7%). Female (aOR=1.44, 95% CI: 1.02 - 2.03), good knowledge of HPV infection (aOR=2.87, 95% CI: 1.82 - 4.53) and good knowledge of HPV vaccine (aOR=18.52, 95% CI: 10.52 - 32.61) were the predictors of HPV vaccine acceptance.

**Conclusion:**

prior to the introduction of HPV vaccine into the routine immunization of Abia State, Nigeria, most parents surveyed indicated that they would accept HPV vaccine for their female adolescents. Sex, knowledge of HPV infection and HPV vaccine were the independent predictors of parental acceptance of HPV vaccine. We recommend the raising of awareness campaign on HPV vaccine benefits and safety, prioritizing healthcare workers and the social media as the major channels of communication to support HPV vaccination.

## Introduction

As of 2020, in Nigeria, 12,075 new cases of cervical cancer and 7,968 deaths were reported [1]. It is the second most frequently diagnosed carcinoma in Nigeria amongst women. The Federal Government of Nigeria, with support from Gavi, the Vaccine Alliance, launched the HPV vaccine implementation on the 24^th^ of October 2023 targeting girls aged 9-14 years with a single dose of quadrivalent HPV vaccine (Gerdasil 4®. Merck, Sharpe & Dohme). It was a phased rollout commencing with 16 States in 2023, and the remaining 21 States to implement in the middle of 2024. This is progress towards achieving one of the World Health Organization (WHO) targets for the elimination of cervical cancer. Previous studies have reported a lower uptake of the HPV vaccine compared to other vaccines administered during adolescence [2,3]. According to Nigeria Demographic and Health Survey (NDHS) 2018, the median age at first intercourse for women within the age group of 20-49 was 18.8 years in Abia State [4]. Earlier onset of coitarche increases susceptibility to HPV infection and transmission to their sexual partners, resulting in an increase of cancer risk [5]. Furthermore, poor uptake contributes hugely to the non-attainment of SDG Goal 3 and reduces herd immunity [6], aiding the spread of the virus within the communities and contributing to the burden on the healthcare system and resources.

In Nigeria, low HPV vaccine acceptance has been attributed to conspiracy theories and myths concerning vaccination amongst parents and caregivers [7]. Other barriers reported by parents include concerns about side effects, cost of the vaccine, low awareness, and fear of sexual promiscuity after the vaccination for protection [8-10]. The lack of integration of parents and caregivers into the vaccination drive contributes to the barriers to vaccine adoption [11]. There is a need to understand the drivers of parental decision-making in accepting the HPV vaccine for their female adolescents. Our findings will contribute to the design of purposeful communication strategies, leveraging the drivers of parental acceptance to eliminate barriers to a successful HPV vaccine implementation. This research aimed to determine the prevalence and identify the predictors of parental acceptance of HPV vaccines for female adolescents prior to HPV vaccine implementation in Abia State, Nigeria.

## Methods

**Study design and setting:** a community-based cross-sectional descriptive study was conducted among parents of female adolescents in Abia State from August to September 2023. Abia State is one of the States in the southeastern geopolitical zone of Nigeria. The 2023 projected total population for the State was 4,457,560. HPV implementation in Nigeria was done in a phased approach and Abia State was one of the phase 1 States to implement the HPV vaccine following the national launch on the 24^th^ October, 2023. The conclusion of the 5-day Multi-Age Cohort (MAC) campaign targeting girls aged 9-14 years in the State initiated the process of HPV vaccine routinization. Over 90% of eligible girls 9-14 years in the State are enrolled in schools. Prior to the introduction, HPV vaccine was not available in any health facility.

**Study participants:** these were parents with female adolescents residing in Abia State. The inclusion criteria included guardians of eligible girls with a decision-making role concerning health and vaccination and parents or guardians who are permanent residents (who had at least lived for 6 months before the survey). Those with severe illnesses that could interfere with the interviewing process were excluded.

**Sampling technique:** we recruited 1,030 respondents using the multistage sampling technique for this survey. In the first stage, simple random sampling (lottery method) was used to select six Local Government Areas (LGAs) out of a total of seventeen LGAs. In stage two, the wards in each selected LGA were enlisted, and two wards were selected in each LGA using balloting. The sample size was proportionally allocated to the size of the selected wards. A census of all households was made across the 12 selected wards. Stage three, following the enumeration of the households in each ward, which served as the sampling frame, random numbers generated using OpenEpi were used to select one eligible respondent from each household. If more than one parent was present, random sampling was used to select one of them. A maximum of three revisits were made to households where the respondents were absent during data collection.

### Operational definitions

**Parental acceptance of HPV vaccine:** the proportion of respondents who were willing to accept the vaccination of their eligible female adolescents with the HPV vaccines when available.

**Measurement of variables:** the dependent variable of the study is parental acceptance of HPV vaccination, which was assessed using the question, “Are you willing to accept the vaccination of your daughter with the HPV vaccine when it is available?” A score of 1 was assigned to ‘yes’ and 0 to ‘no’ responses. The independent variables in this study included age, sex, religion, denomination, monthly income, marital status, educational level, employment status, occupational status, and sources of information about the HPV vaccine, reasons for acceptance/refusal of the vaccine, and sources of preferred recommendation by the respondents. Knowledge of HPV infection and HPV vaccines was assessed with 10 and 8 questions, respectively. A correct response was scored as 1 point, while an incorrect or unmentioned correct response was scored as 0. The minimum and maximum possible scores were 0 and 22 (knowledge of HPV infection), 0 and 15 (knowledge of HPV vaccine), respectively. Using the mean score as a cutoff, knowledge was categorized into good knowledge (scores above the mean) and poor knowledge (scores below the mean score).

**Sample size determination:** the sample size was calculated using the formula:


$$\frac{{\left(Z_{\alpha }+Z_{\beta }\right)}^{2}pq}{d^{2}}$$

Where, Z_α_ represents the standard normal deviate for an alpha level of significance set at 1.96, corresponding to a 95 percent confidence interval. Z_β_ is the standard normal deviate for the power of the study, set at 80%, which corresponds to 0.84. A parental acceptance of 62.8% based on a previous study conducted in the Federal Capital Territory, Nigeria, was used as the estimated prevalence (p) [12]. The precision (d) was set at 5%. Accounting for a non-response rate of 20%, the final minimum sample size was calculated to be 916 respondents.

**Data collection tool and methods:** a structured interviewer-administered questionnaire, adapted and modified from previous studies [13-15] was used to collect information from the respondents. The reliability and validity of the questionnaire were assessed using content and face validity techniques. The questionnaire consisted of four sections, preceded by an introductory part outlining the background of the study, its benefits, and assurances of confidentiality regarding respondents’ responses. Section A contained the sociodemographic characteristics of the respondents, such as age, marital status, religion, denomination, education and income status, and number of living children. Sections B and C comprised questions on the sources of information for HPV vaccine, knowledge of HPV infection and the HPV vaccine. Sections D addressed questions on the acceptability of HPV vaccination and the reasons for acceptance or refusal of HPV vaccines. To establish face validity, a pretest was done in Aba South (a non-sampled LGA) using 5% of the calculated sample size. The result of the pretest was used to improve the clarity and wording of the questionnaire as well as the logical sequence of the questions. Efforts were made to ensure that the population to be used for the pretest had similar sociodemographic characteristics with the study population of the study sites. The finalized questionnaire was transferred into tablets and Android phones using the ODK Collect and synced to a central database (Kobo Collect Server). The Igbo translated version was also available for respondents who did not understand English. A total of six research assistants were recruited for the study and were responsible for administering the questionnaires to the respondents. They were trained by the principal investigator over two days on research ethics and the interview process. The estimated time for an interview section was 10 to 15 minutes.

**Statistical analysis:** data was downloaded from the Kobo Collect Server and cleaned. The data were coded and analyzed using SPSS version 26. Univariate analysis was used to generate frequency tables and proportions. Bivariate analysis was used to test for associations between the independent variables and parental acceptance of HPV vaccine. P values <0.05 were considered significant. Backward stepwise multivariable logistic regression was used to determine the independent predictors of parental acceptance of HPV vaccine. Factors included in the final model were those with P values <0.2 at the level of bivariate analysis. The adjusted odds ratios and 95% confidence intervals were obtained at significance level of 5%. Appropriate charts were used for further representation of the data.

**Ethical consideration:** ethical clearance was obtained from the Research Ethical Committee of Federal Medical Centre, Umuahia, Abia State with the reference number FMC/QEH/G.596/Vol.10/669. Respondents´ privacy and confidentiality were maintained. They were also made to understand that their participation was voluntary, and informed written consent was obtained before administering the questionnaire to the respondents. Data security was assured by storing the data on a passworded computer which was only accessible to the principal investigator.

## Results

**Sociodemographic characteristics of respondents:** a total of 1,016 respondents participated in the study out of 1,030 recruited giving a response rate of 98.6%. The majority (53.3%) of the respondents reside in urban areas. Most of the respondents (35.9%) were within the age group of 40-49 years. The mean age was 42.2 ± 10.5 years among the respondents. Two-thirds of the respondents (66.7%) were females (parents/guardians). Most of the respondents had attained tertiary education (52.2%) and married (69.7%). Christianity was the major religion (97.5%) with 40.5% of all the respondents belonging to the Pentecostal denomination. Almost half of the respondents (49.9%) were self-employed, followed by salary earners (33.2%) and unemployed (16.9%). A greater proportion of the respondents (42.6%) reported an average monthly household income of less than ₦50,000 (approximately 33.3 USD), while only a few (0.6%) earned more than ₦1,000,000 (approximately 665.3 USD) per month. Most of the respondents (45.2%) had 3-4 children (Table 1)

**Table 1:** socio-demographic characteristics of the respondents (n=1016)

Variables	Frequency	Percentage (%)
**Type of residence**		
Urban	541	53.3
Rural	475	46.7
**Age (years)**		
≤ 29	155	15.2
30-39	261	25.6
40-49	365	35.9
50-59	169	16.7
≥ 60	66	6.6
Mean ± SD	42.2 ± 10.5	
**Sex**		
Female	677	66.7
Male	339	33.3
**Educational status**		
No formal education	29	2.8
Primary	80	7.8
Secondary	378	37.2
Tertiary	529	52.2
**Marital status**		
Single	95	9.4
married	708	69.7
Cohabiting	18	1.8
Divorced	36	3.5
Widowed	95	9.4
Separated	64	6.2
**Religion**		
Christianity	991	97.5
Islam	5	0.5
African traditional religion	19	1.9
Others	1	0.1
Denomination*		
Pentecostal	400	40.5
Orthodox	300	30.3
Catholic	284	28.7
Others	5	0.5
**Occupational status**		
Civil servant	323	31.8
Businessman/woman	476	46.9
Student	54	5.3
Unemployed	112	11.0
Others	51	5.0
**Employment status**		
Salary earner	337	33.2
Self employed	507	49.9
Not employed	172	16.9
**Monthly household income (Naira ₦)**		
None	253	24.9
<50,000	433	42.6
50,000 - 100,000	268	26.3
>100,000 - 1,000,000	57	5.6
>1,000,000	5	0.6
**Number of children**		
≤2	311	30.6
3-4	459	45.2
>4	246	24.2

* n=989

**Figure 1:**
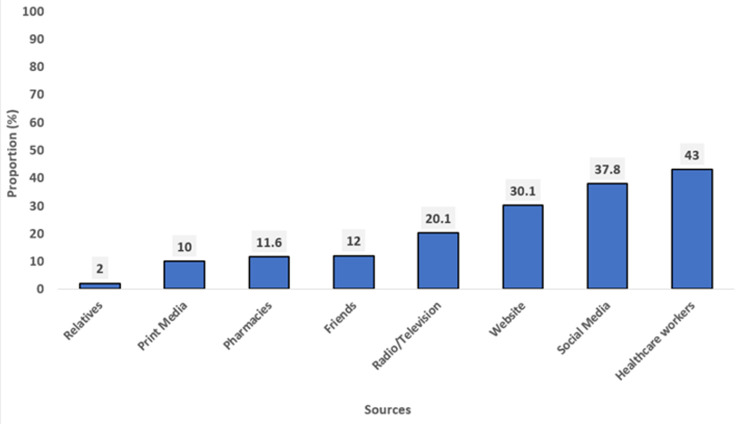
sources of information on HPV vaccine among the respondents

**Sources of information on HPV vaccine among the respondents:** health workers were the major source of information (43%), followed by social media (37.8%) and Website (30.1%). Print media, (10%) and relatives, (2%) were the least sources of information of HPV vaccine (Figure 1).

**Figure 2:**
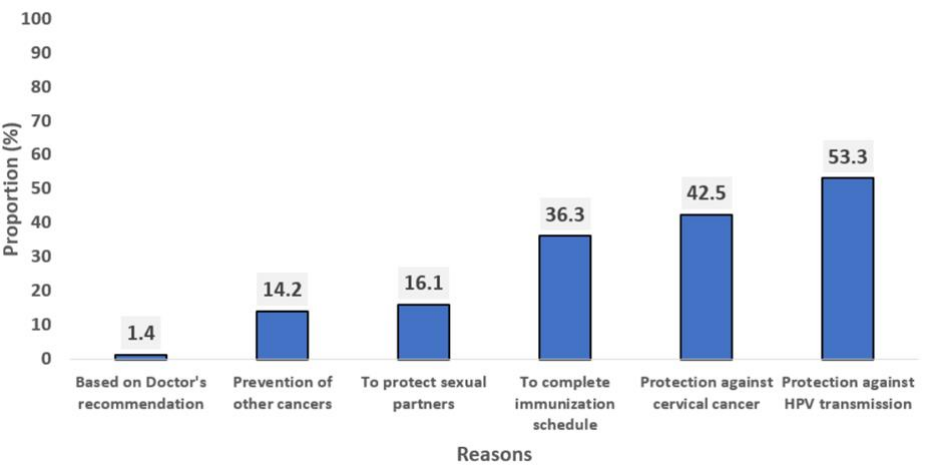
reasons for acceptance of HPV vaccine for their female adolescents among the respondents (n=640)

**Reasons for parental acceptance of HPV vaccine for their female adolescents:** the prevalence of parental HPV vaccine acceptance was 63.0% (95% CI: 59.8 - 66.0). Among these respondents who expressed willingness to accept the vaccination of their female adolescents, protection against HPV transmission was their major reason (58.3%) for HPV vaccine acceptance. Other notable reasons for acceptance included protection against cervical cancer (47.5%) and to complete their immunization schedule (41.3%) (Figure 2).

**Figure 3:**
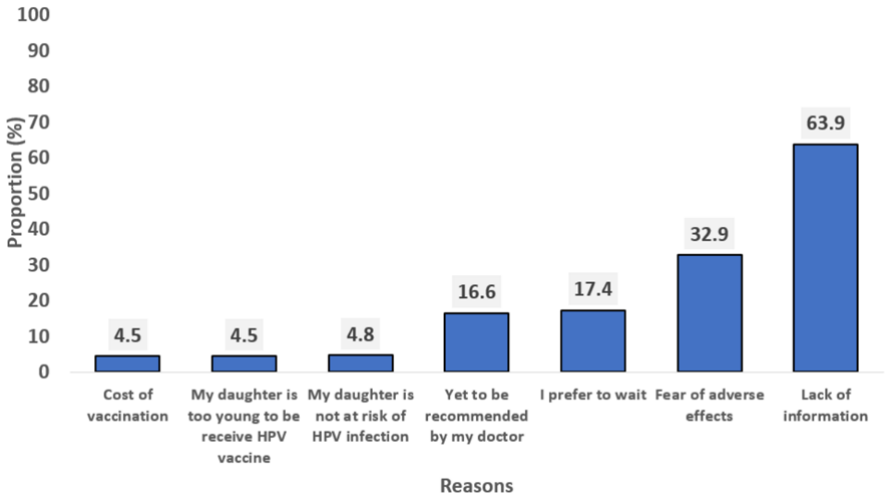
reasons for non-acceptance of HPV vaccine for their adolescents among the respondents (n=376)

**Reasons for parental non-acceptance of HPV vaccines for their female adolescents:** for the respondents who declined willingness to accept the vaccine, most of them (63.9%) cited lack of information as the main reason for not accepting HPV vaccine for their girls. However, other reasons included fear of adverse effects (32.9%) and delayed decision making for vaccine acceptance (17.4%). Furthermore, the least reasons mentioned included the child being too young to receive the vaccine (4.5%) and cost of vaccination (4.5%) (Figure 3).

**Figure 4:**
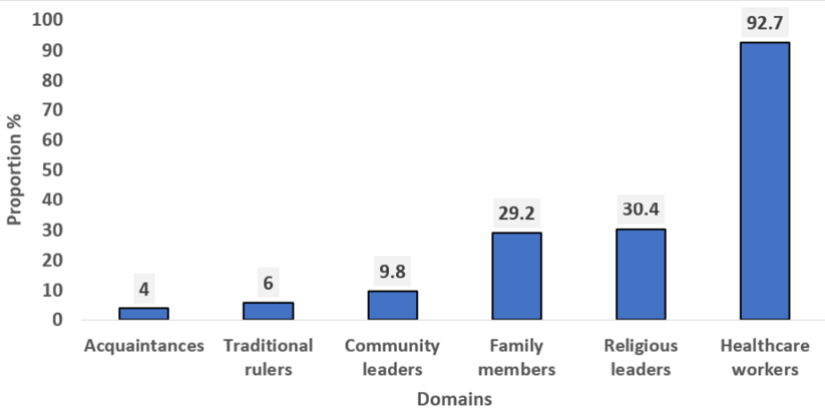
preferred sources of recommendations for parental HPV vaccine acceptance (n=1016)

**Preferred sources of recommendations for parental HPV vaccine acceptance:** among all respondents, the most preferred source of HPV vaccine recommendation was healthcare workers (92.7%), followed by the religious leaders (30.4%), while the least mentioned was their acquaintances (4%) (Figure 4).

**Independent predictors of parental acceptance of HPV vaccine:** in the bivariate logistic regression model, sex (parents/guardians), educational status, religion, employment status, HPV infection knowledge and HPV vaccine knowledge were significantly associated with HPV vaccination acceptance. The female respondents had 61% higher odds of accepting the vaccine compared to their male counterparts (COR= 1.61, 95% CI; 1.22 - 2.12). Attaining a tertiary level of education had higher odds of HPV vaccine acceptance, (COR= 2.18, 95% CI; 1.67 - 2.86). Similarly, Christians had over a threefold higher likelihood of accepting the vaccine (COR= 3.74, 95% CI; 1.50 - 10.10). Also, salary earners were more likely to accept the HPV vaccine (COR= 2.16, 95% CI; 1.45 - 3.20). Furthermore, respondents with good knowledge of HPV infection and HPV vaccine had higher odds of HPV vaccine acceptance. (COR= 13.8, 95% CI; 9.51 - 20.29) and (COR= 37.19, 95% CI; 22.34 - 61.35) respectively. The multivariate logistic regression analysis identified female (aOR= 1.44, 95% CI; 1.02 - 2.03), good knowledge of HPV infection (aOR= 2.87, 95% CI; 1.82 - 4.53) and good knowledge of HPV vaccine (aOR= 18.52, 95% CI; 10.52 - 32.61) as the predictors of HPV vaccine acceptance among the respondents (Table 2).

**Table 2:** independent predictors of parental acceptance of HPV vaccine

Variables	HPV vaccine Acceptance		COR (95% CI)	P value	aOR (95% CI)	P value
	Yes (n%)	No (n%)				
All	640 (63.0)	376 (37.0)				
**Age (years)**						
< 30	275 (66.3)	140 (33.7)	1.27 (0.97-1.66)	0.074	0.96 (0.69-1.35)	0.833
≥ 30	365 (60.7)	236 (39.3)	1		1	
**Sex**						
Female	452 (66.8)	225 (33.2)	1.61 (1.22-2.12)	0.004*	1.44 (1.02-2.03)	0.038*
Male	188 (55.5)	151 (44.5)	1		1	
**Type of residence**						
Rural	306 (64.4)	169 (35.6)	1.12 (0.86-1.46)	0.367	----	---
Urban	334 (61.7)	207 (38.3)	1			
**Educational status**						
Tertiary	379 (71.6)	150 (28.4)	2.18 (1.67-2.86)	0.001*	1.04 (0.74-1.44)	0.839
Below tertiary	261 (53.6)	226 (46.4)	1		1	
**Marital status**						
Not married	190 (65.3)	101 (34.7)	1.14 (0.85-1.54)	0.336	----	--
Married	450 (62.1)	275 (37.9)	1			
**Religion**						
Christianity	632 (63.8)	359 (36.2)	3.74 (1.50- 10.10)	0.012*	----	--
Others	8 (32)	17 (68)	1			
Denomination						
Protestants	460 (65.2)	245 (34.8)	1.24 (0.92-1.66)	0.135	----	--
Catholic	171 (60.2)	113 (39.8)	1			
**Employment status**						
Salary earner	255 (76.1)	80 (23.9)	2.16 (1.45-3.20)	0.001*	----	--
Self employed	283 (55.5)	227 (44.5)	0.84 (0.59-1.20)	0.343	----	--
Unemployed	102 (59.6)	69 (40.4)	1			
**Monthly household income (Naira ₦)**						
< 50,000	433 (63.1)	253 (36.9)	1.15 (0.65-2.02)	0.591	----	--
50,000 - 100,000	170 (63.4)	98 (36.6)	1.17 (0.63- 2.13)	0.581	----	--
> 100,000	37 (59.7)	25 (40.3)	1			
**HPV knowledge status**						
Good	402 (90.7)	41 (9.3)	13.8 (9.61-19.82)	< 0.001*	2.87 (1.82-4.53)	< 0.001*
Poor	238 (41.5)	335 (58.5)	1		1	
**HPV vaccine knowledge status**						
Good	417 (95.9)	18 (4.1)	37.19 (22.54-61.35)	< 0.001*	18.52 (10.52-32.61)	< 0.001*
Poor	223 (38.4)	358 (61.6)	1		1	

* p values < 0.05 are considered significant; HPV: human papillomavirus

## Discussion

This study determined the prevalence of HPV vaccine acceptance and its predictors among the parents and caregivers of female adolescents in Abia State, Nigeria. Three out of five respondents expressed willingness to accept the HPV vaccine for their female adolescents. The major reason for acceptance of HPV vaccine was to protect the female adolescents from HPV infection. Lack of information, followed by fear of side effects were the major reasons for refusal of the vaccine. The most preferable source of HPV vaccine recommendation were the healthcare workers. The predictors included being a female (parent/guardian), having good knowledge of HPV infection and HPV vaccine.

In this study, 63% of the respondents were willing to vaccinate their female adolescents with the HPV vaccine when available. This concurs with a report of 62.8% acceptance of the vaccine as documented in a study conducted in north-central Nigeria [ 12]. Studies in China and Poland observed lower values of 55% and 40.8% respectively compared to the findings in our study [16,17]. However, higher values of 81.8%, 94.3%, 93.7% and 79.9% have been documented in recent studies done in Nigeria, Ethiopia, Brazil, India and UAE (United Arab Emirates) respectively [13,14,18-20]. These discrepancies in figures could be attributed to variations in the timing of HPV vaccine introduction for the different countries, as well as individual differences in vaccination behaviours and beliefs. This study was conducted before the HPV vaccine implementation in Nigeria, with the national launch held on the 24^th^ of October 2023. There is a need to develop and implement effective communication strategies, especially in the phase 2 States as well as other countries with similar sociocultural settings, yet to roll out the HPV vaccines. This will increase the level of awareness of the vaccine in the community, address vaccine distrust/fears, and boost vaccine confidence.

The major source of information for the HPV vaccine among the respondents was the healthcare providers. This corroborates the finding of a previous study in Nigeria where doctors were the most prevalent source of information [21]. Other common sources that have been noted in other studies include the media [21,22] and radio/television [23]. Healthcare workers are trained professionals with accurate and up-to-date information on HPV vaccines. Seeking information from them ensures reliable guidance for specific health concerns, promotes informed decisions, and fosters trust in the vaccination process, thereby promoting vaccine uptake.

Lack of information was the leading reason for HPV vaccine refusal, followed by fear of adverse effects. This finding is consistent with previous studies in Nigeria which highlight the influence of low levels of awareness and concerns of adverse effects on vaccination decisions [8,10]. In other studies, the need for further research on the new vaccine was a prevalent reason for the refusal of vaccines [24,25]. On the other hand, most respondents who accepted the HPV vaccine ranked the protection of the girl child as the main reason to accept the vaccination. This is concurrent with the finding of a study indicating that people are prone to accepting the HPV vaccine if they believe that it can prevent the transmission of HPV [26]. Conversely, in a study from Califonia, healthcare providers´ recommendation was the main reason for HPV vaccine acceptance [27].

The most preferred source of HPV vaccine recommendation for parental acceptance was healthcare workers. This finding aligns with existing literature on the factors associated with parental uptake of HPV vaccine for their children [22,28]. Healthcare workers are an important source of immunization information. People tend to have trust in the information provided by healthcare workers due to their frequent interactions with them during health-related decision-making [29]. This increases their vaccine confidence, thereby improving their vaccination behaviours [30]. Also, it emphasizes the need for adequate training of the healthcare workers during the HPV vaccine pre-implementation phase to heighten their confidence when sensitizing the parents and caregivers of the adolescents.

Females (parents/guardians) had higher odds of HPV parental acceptance compared to the males. This corroborates the findings documented in some published research [31,32]. However, this finding contrasts with a recent study in Ethiopia, where men were more likely to accept the HPV vaccine for their eligible daughters compared to the mothers [33]. Women may be more inclined to accept the vaccine for their daughters compared to men because they have a strong instinct towards protecting their girls and thereby encouraging them to take preventive measures against these potential risks. Additionally, most awareness campaigns focus solely on cervical cancer, reinforcing the perception that the vaccine is mostly relevant to females. There is a need for male involvement in the awareness campaigns to highlight the broader spectrum of HPV-related infections so that both sexes appreciate the importance of preventing the transmission of HPV. In this current study, respondents with good knowledge of HPV infection and HPV vaccine were more likely to accept the HPV vaccine for their female adolescents compared to their counterparts. This is similar to the findings of studies done in Ethiopia and China [13,34,35].

High knowledge of HPV infection can enhance HPV vaccine acceptability by fostering a deeper understanding of its associated health risks. With comprehensive information, people are more likely to recognize the importance of prevention through vaccination. Similarly, good knowledge of the HPV vaccine provides accurate information about vaccine safety and effectiveness, benefits, occurrence, and frequency of potential side effects. This helps boost trust and confidence, encouraging people to accept vaccination.

## Conclusion

The acceptance of the HPV vaccine was moderately high, as three out of five respondents expressed a willingness to get their female adolescents vaccinated. Sex (parents/guardians), knowledge of HPV infection, and HPV vaccine were the independent predictors of parental acceptance of the HPV vaccine. The most prevalent source of information for the HPV vaccine was the healthcare providers. Also, the recommendation of the HPV vaccine from healthcare workers exerted the highest influence on accepting the HPV vaccine. The reasons for HPV vaccine acceptance, as well as reasons for the refusal of the HPV vaccine amongst the respondents, were documented in this study. There is a need to prioritize the improvement of knowledge of HPV infection and vaccines, and to ramp up healthcare workers´ recommendations to increase parental acceptance of the vaccine. We recommend an awareness campaign on the HPV vaccine, prioritizing healthcare workers and social media as the major channels of communication and focusing on mothers and female caregivers as a crucial target audience. We also recommend men's involvement in all the activities targeted at increasing the willingness to accept the HPV vaccine. Some of the limitations of this study included the possibility of self-reported bias since the responses were subjective. Also, the temporality and causal inference could not be established in the context of the study design type. However, the details about the purpose of this study were well communicated to the respondents, and they were assured of their confidentiality. Nevertheless, the major strength of this study lies in the fact that it was conducted before the implementation of HPV vaccination in Abia State. The study, therefore, provided a pre-assessment of parental willingness to vaccinate their daughters amid the ongoing social mobilization efforts to create demand for the HPV vaccine. Furthermore, the sample size was large enough and a good representative of the study sites, aiding the generalizability of our findings.

### What is known about this topic

- Cervical cancer is the fourth-commonest cancer worldwide and the second-leading cause of cancer-related deaths among women;
- Globally, sub-Sahara Africa (SSA) accounts for most cases of cervical cancer;
- Vaccination against HPV is an effective preventive intervention against HPV infections.

### What this study adds

- This study provides baseline data on parental willingness to accept the HPV vaccine before its implementation in the State, informing future vaccination strategies;
- It provided the factors that should be prioritized to increase the acceptability of the HPV vaccine during the campaign and subsequent routinization of the HPV vaccine in the State.
